# Insights into Plant Sensory Mechanisms under Abiotic Stresses

**DOI:** 10.3390/plants13141907

**Published:** 2024-07-10

**Authors:** Songsong Jin, Mengting Wei, Yunmin Wei, Zhonghao Jiang

**Affiliations:** 1 College of Life Sciences and Oceanography, Shenzhen University, Shenzhen 518060, China; jsssnd@szu.edu.cn (S.J.); 2100252009@email.szu.edu.cn (M.W.); weiym1024@163.com (Y.W.); 2 College of Physics and Optoelectronic Engineering, Shenzhen University, Shenzhen 518060, China

**Keywords:** abiotic stresses, crop productivity, sensors, Ca^2+^-permeable channels, RLKs, sphingolipids

## Abstract

As sessile organisms, plants cannot survive in harmful environments, such as those characterized by drought, flood, heat, cold, nutrient deficiency, and salt or toxic metal stress. These stressors impair plant growth and development, leading to decreased crop productivity. To induce an appropriate response to abiotic stresses, plants must sense the pertinent stressor at an early stage to initiate precise signal transduction. Here, we provide an overview of recent progress in our understanding of the molecular mechanisms underlying plant abiotic stress sensing. Numerous biomolecules have been found to participate in the process of abiotic stress sensing and function as abiotic stress sensors in plants. Based on their molecular structure, these biomolecules can be divided into four groups: Ca^2+^-permeable channels, receptor-like kinases (RLKs), sphingolipids, and other proteins. This improved knowledge can be used to identify key molecular targets for engineering stress-resilient crops in the field.

## 1. Introduction

As sessile organisms, plants are unable to move to favorable environments and must trigger numerous responses to survive when they are challenged by adverse environmental conditions, such as drought, flooding, heat, cold, nutrient deficiency, and salt or toxic metal stress. These abiotic stresses impair plant development and reproduction, leading to decreased crop productivity [1,2,3,4]. Therefore, understanding the molecular basis of how plants perceive and respond to abiotic stresses is critical for food security worldwide.

Relative to the identification of downstream cellular signaling pathways and physiological responses to these abiotic stresses, research on plant sensory mechanisms has long been lacking. In nature, multiple abiotic stresses often occur simultaneously, such as flood stress, which simultaneously imposes hypoxia, photosynthesis reduction, and mechanical stress [5,6]. Furthermore, even an individual abiotic stress can trigger more than one stress; for example, salt stress can induce three stresses: osmotic stress, ionic stress, and secondary stress (such as oxidative stress) [7,8]. This complexity of environmental abiotic stresses makes it difficult to identify abiotic stress sensors in plants. As a pioneer, the primary abiotic stress sensors should detect the occurrence of the pertinent stressor at an early stage and convert external stimuli to cellular signals. It is difficult to demonstrate that a biomolecule directly senses stress. The identification of most putative stress sensors is based on indirect approaches. For instance, impairing the function of sensors is expected to affect the levels of second messengers such as calcium (Ca^2+^), reactive oxygen species (ROS), nitric oxide (NO), and phospholipids [8,9,10]. To identify abiotic stress sensors, an early readout of the signaling pathway should be chosen to avoid complications from downstream signaling interactions and integration. Rapid changes in the intracellular concentration of free Ca^2+^ constitute one of the earliest signaling events in plants in response to external stressors [11,12]. A transient increase in cytosolic Ca^2+^ concentrations ([Ca^2+^]) is a common theme of abiotic stress sensing and is detected by bioluminescence-based aequorin technology, in which the aequorin system is used to detect Ca^2+^ signaling induced by abiotic stimuli [11,12]. This spatiotemporally defined increase in the cytoplasmic Ca^2+^ concentration is caused by the combined effects of Ca^2+^ influx and efflux mechanisms, which depend on numerous plasma-membrane-localized Ca^2+^-permeable channels or transporters [9,13,14,15]. In addition, different abiotic stresses activate different Ca^2+^ signature, including tissue-specific and stress-specific differences in Ca^2+^ peak amplitude, oscillation pattern, and Ca^2+^ wave propagation in cells [8,9,10].

Abiotic stress signals can simultaneously impact all parts of cells and are perceived at various locations of the cells [16]. Biological membrane-anchored proteins, such as Ca^2+^-permeable channels and receptor-like kinases (RLKs), are regarded as candidates for abiotic stress sensors. For example, plasma-membrane-located cyclic-nucleotide-gated calcium channels (CNGCs) can monitor the fluidity of cellular membranes and sense extreme temperature stress [17,18,19]. The hydrogen-peroxide-induced Ca^2+^ increases 1 (HPCA1), as a leucine-rich repeat receptor kinase, is a representative of RLKs and functions in sensing oxidative stress [10]. Membrane lipids interact with membrane proteins and regulate their fuctions. Sphingolipids are a lipid composition of plasma membrane and modulate cellular signal transduction events [20]. Recently, glycosylinositol phosphoceramides (GIPCs) have been identified as a salt stress sensor [8]. In addition, non-membrane proteins located in the nucleus and cytosol are also found to participate in sensing abiotic stresses and are dependent on protein conformation changes, such as EARLY FLOWERING 3 (ELF3) [21] and phytochrome B (phyB) [22,23].

In this review, we summarize and discuss recent studies in the field of plant sensing of abiotic stresses. Numerous biomolecules have been found to participate in abiotic stress sensing and function as abiotic stress sensors in plants. Based on their molecular structure, these biomolecules can be divided into four groups: Ca^2+^-permeable channels, RLKs, sphingolipids, and other proteins. Here, we focus on initial abiotic stress signal perception and do not elaborate on the roles of the above-mentioned biomolecules in other bioprocesses, such as plant growth and development and response to biotic stress, which have been discussed in detail in several recent reviews [16,24,25,26,27,28]. The analysis of the above-mentioned biomolecules based on molecular structure provides a new research direction for uncovering more abiotic stress sensors in plants. Understanding the mechanisms by which plants sense stressful environments will provide information for genetically engineering abiotic stress-tolerant crops to meet the demand for increased food production for an increasing world population.

## 2. Classification of Biomolecules Participating in Abiotic Stress Sensing

### 2.1. Ca^2+^-Permeable Channels

Changes in the cytosolic Ca^2+^ concentration are regulated by Ca^2+^-permeable channels. There are more than 40 genes encoding putative Ca^2+^ channels in the Arabidopsis genome and more than 20 genes encoding putative Ca^2+^ channels in the rice genome, some of which may function in abiotic stress sensing [14,29,30].

#### 2.1.1. Hyperosmolarity-Gated Calcium-Permeable Channel Family of Proteins (OSCAs)

In Arabidopsis, the plasma-membrane-localized OSCA1 was identified as the first putative osmosensor using a Ca^2+^ imaging-based forward genetic screen (Table 1) [9]. The *osca1* mutant was isolated from EMS-mutagenized aequorin-expressing Arabidopsis seedlings, demonstrating decreased Ca^2+^ accumulation in guard cells and root cells when exposed to sorbitol but not in response to H_2_O_2_ or ABA. Under osmotic stress conditions, reduced primary root length and leaf area and attenuated transpiration were observed in *osca1* seedlings [9]. *AtOSCA1* is a founding member of a gene family that includes at least 15 ion channels with similar structural features in Arabidopsis, and the OSCA family may mediate the sensing of hyperosmotic conditions [9,31]. Recently, OSCA2.1 and OSCA2.2 were identified as essential hypo-osmosensors in Arabidopsis. The double-loss-function mutant *osca2.1/osca2.2* defected in pollen germination and was sensitive to hypo-osmolarity. OSCA2.1 and OSCA2.2 perceived extracellular water status and converted it into Ca^2+^ spiking in pollen [32]. In rice, a genome-wide survey found 11 genes in the entire *OSCA* family, containing a conserved DUF221 domain [31]. In maize, 12 *ZmOSCAs* were identified from the genome database of maize [33]. The expression profiles of *OSCAs* are variant in different tissues and under diverse abiotic stresses [33,34]. Subsequently, the cryo-electron microscopy (cryo-EM) structures and functions of the OSCA1 homologs CALCIUM PERMEABLE STRESS-GATED CATION CHANNEL1 (AtCSC1A/AtOSCA1.2), AtOSCA1.1, AtOSCA3.1, and OsOSCA1.2 were analyzed, providing a model of how they could mediate hyperosmolality sensing and transport pathway gating. They share similar protein fold and topology. OSCA channels are dimeric architectures containing 11 transmembrane (TM) helices, associated extracellular loops, intracellular loops, and an intracellular soluble domain [31,35,36,37,38]. However, the gating mechanisms of OSCAs remain poorly understood and need to be determined.

#### 2.1.2. CNGCs

The fluidity of cellular membranes is related to Ca^2+^ influx and influenced by extreme temperatures, such as decreases caused by cold stress or increases caused by heat stress, which may be sensed by plasma membrane CNGCs (Table 1) [48]. CNGCs have been reported to play important roles in plant thermal sensing and acquired thermotolerance. In rice, *OsCNGC9* confers enhanced chilling tolerance by mediating cold-induced Ca^2+^ influx. The *cds1* (*cell death and susceptible to blast 1*), a loss-of-function mutant of *OsCNGC9*, is more sensitive to chilling shock. OsCNGC9 is phosphorylated and activated by OsSAPK8, a homolog of AtOST1, to trigger cytoplasmic calcium elevation. In addition, OsDREB1A positively regulates transcriptional expression of *OsCNGC9* [15]. OsCNGC14 and OsCNGC16 play vital roles in response to heat stimulation as well as low-temperature stress by impacting cytosolic calcium increase in rice. Their loss-of-function mutants, *cngc14* and *cngc16*, are generated by genome editing and have more withered and yellower leaves, lower survival rates, higher H_2_O_2_ accumulation, more cell damage, and stronger defect in calcium signal compared to the wild-type *cv Nipponbare* plants under a heat treatment or chilling treatment [17]. Furthermore, their homologs, AtCNGC2 and AtCNGC4, in Arabidopsis lead to chilling tolerance and acquired thermotolerance by regulating cytosolic Ca^2+^ [17,18]. Two loss-of-function mutants of *AtCNGC2* and *AtCNGC4*, *cngc2* and *cngc4*, display reduced hypocotyl elongation, rosette growth, and fresh weight under chilling conditions [17]. Disruption of *AtCNGC2*, *AtCNGC4*, and *AtCNGC6* results in a hyper-thermosensitive phenotype, such as a defect in heat-induced increases in cytosolic Ca^2+^, reduced expression of heat shock protein (HSP) genes and abolished thermotolerance [18,19]. CNGCs are reported as putative abiotic stress sensors based on their direct regulation to cytosolic Ca^2+^ concentration in cell, but their activation mechanisms remain to be investigated.

#### 2.1.3. ANNEXIN Proteins (ANNs)

ANNs act as Ca^2+^-permeable transporters and regulate stress-induced cytosolic free Ca^2+^ ([Ca^2+^]_cyt_) elevations in plant response to abiotic stress (Table 1). In Arabidopsis, MYB30, a R2R3-MYB transcription factor, regulates negatively [Ca^2+^]_cyt_ in response to oxidation stress and heat stresses, which depend on the function of ANN proteins [39]. Transcriptional expression of *ANN1* and *ANN4* is repressed when MYB30 binds to their promoters. The single mutant *myb30* is sensitive to MV and heat treatment. However, the triple mutant *myb30 ann1 ann4* displays an attenuated phenotype compared with *myb30* under MV and heat treatment. In addition, the application of LaCl_3_, a calcium channel blocker, can suppress the MV and heat sensitivity of *myb30* [39]. AtANN1 and AtANN4 are also found to mediate cold-induced Ca^2+^ influx and confer enhanced freezing tolerance in Arabidopsis. The loss of function of AtANN1 and AtANN4 exhibits reduced freezing tolerance. The single mutant *atann1*, *atann4-1* and double mutant *atann1 atann4-1* display lower survival rates than the wide type [40,42]. AtANN1 is phosphorylated by the OST1/SnRK2.6 kinase and acts downstream of OST1 in responses to freezing shock. The cascade linking OST1-AtANN1 triggers cold-induced [Ca^2+^]_cyt_ elevation and activates the cold response to acclimate to freezing conditions [40]. Under salt stress conditions, root epidermal net Na^+^ influx in *atann1* is significantly higher than the wild type, while transient [Ca^2+^]_cyt_ increase is significantly lower in *atann1* than the wild type. This phenomenon shows that AtANN1 restricts Na^+^ Influx and positively regulates NaCl-induced Ca^2+^ influx to improve salt stress tolerance [41]. Moreover, AtANN4 plays an important role in plant responses to salt stress by a negative feedback regulatory loop. AtANN4 interacts with the SOS2-SCaBP8 complex to increase salt-induced Ca^2+^ influx and, then, initiates a specific salt-induced calcium signal [42]. ANNs are found to play multifaceted roles in plants; however, knowledge about their functions is still in its infancy. Further research is needed to exploit the potential abiotic stress sensing mechanisms mediated by ANNs in plants.

#### 2.1.4. Glutamate Receptor-like Proteins (GLRs)

Plant GLRs are ligand-gated ion channels and act as Ca^2+^-permeable channels to mediate Ca^2+^ signaling (Table 1) [43,49]. GLRs can perceive environmental stress signals and convert them into specific stress-induced Ca^2+^ elevations that propagate to distant organs, to initiate stress defense responses in the whole plant [50]. In Arabidopsis, there are 20 members in the GLR family [51]. The application of glutamate (Glu) and glycine (Gly) triggers a very large and fast transient spike in [Ca^2+^]_cyt_ accompanied membrane depolarization, suggesting that Glu and Gly participate to control the ligand-mediated gating of calcium in plants [52,53]. The membrane depolarization and associated rise in cytosolic Ca^2+^ triggered by Glu are abolished in *glr3.3-1* and *glr3.3-2*, two loss-of-function mutants, indicating GLR3.3 mediates Glu-triggered Ca^2+^ influx [43]. Additionally, AtGLR3.4 participates in response to abiotic stress stimuli, such as touch, osmotic stress, or cold stress [44]. GLRs are emerging as a novel signaling molecule involved in plant sensory mechanisms under abiotic stresses. Additional research is needed to explore the specific functions and activation mechanisms of GLRs in plant responses to abiotic stresses.

#### 2.1.5. Mid1-Complementing Activity Proteins (MCAs)

MCAs, which are Ca^2+^-permeable mechanosensitive ion channels localized to the plasma membrane, have been identified as mechanosensitive sensors (Table 1). Arabidopsis transgenic lines MCA1ox (overexpressed *AtMCA1* cDNA) roots accumulate a greater extent of Ca^2+^ than wide-type and *mca1-null* (a T-DNA insertion mutant of *AtMCA1*) roots, suggesting that *AtMCA1* promotes Ca^2+^ uptake in roots. Under hypo-osmotic stress or treatment of the anionic amphipath trinitrophenol (TNP), generating membrane distortion, MCA1ox seedlings show greater [Ca^2+^]_cyt_ increase compared with wide-type and *mca1-null* seedlings. Growing on a lower (harder) medium containing 1.6% agar covered with an upper (softer) medium containing 0.8% agar, only the primary roots of *mca1-null* seedlings cannot penetrate a harder agar medium from a softer one. These studies indicate that MCA1 promotes Ca^2+^ influx upon plasma membrane distortion, which leads to mechanosensing and soil hardness sensing [13]. MCA2 has been identified as a Ca^2+^-permeable mechanosensitive channel and directly activated by sensing membrane tension. MCA1 (1-173) and MCA2 (1-173), the N-terminal 173 residues of MCA1 and MCA2, are able to mediate Ca^2+^ influx and maintain mechanosensitivity [45]. In addition, MCA1 and MCA2 are involved in the process of sensing gravity signals in plants. Hypergravity stress induces the expression of *MCA1* and *MCA2*. The degree of hypergravity suppressing the elongation growth of hypocotyls in *mca-null* mutants is lower than that in the wide type. The overexpression of *MCAs* leads the plant to become sensitive to increased gravity [46]. The gravistimulation-induced very slow Ca^2+^ increase is defective in *mca1-null* mutants [47]. However, the direct involvement of MCAs in plant sensory mechanisms under abiotic stresses is still unclear and needs to be investigated.

### 2.2. RLKs

In Arabidopsis, the largest protein family, RLKs, contains 610 members, including 417 receptor kinases; the other 193 members lack the signature signal sequence and/or transmembrane sequence [54,55]. Multiple lines of evidence suggest that plant RLKs play a vital role in perceiving external signals under abiotic stress. Several transmembrane RLKs have been reported to play vital roles in abiotic stress sensing, including *Catharanthus roseus* receptor-like kinase 1-like (*Cr*RLK1L) family protein FERONIA (FER), THESEUS1 (THE1), HERCULES1 and 2 (HERK1 and 2), MALE DISCOVERER1-INTERACTING RECEPTOR LIKE KINASE 2/LEUCINE-RICH REPEAT KINASE FAMILY PROTEIN INDUCED BY SALT STRESS (MIK2/LRR-KISS), HPCA1, root meristem growth factor receptors and plant elicitor peptide receptors (RGFRs and PEPRs), and the aluminum ion sensor Al Resistance1 (ALR1) [7,10,56,57,58,59,60,61,62,63,64] (Table 2).

#### 2.2.1. Cell Wall Integrity Sensors

Salt stress and drought stress both induce hyperosmotic stress in plant cells, leading to the loss of turgor pressure, plasmolysis, and detachment of the plasma membrane from the cell wall. The integrity of the cell wall can be monitored by plasma-membrane-located RLKs. *Cr*RLK1Ls, wall-associated kinases (WAKs), and LRR-RKs are putative sensors of cell wall integrity. Here, FER, THE1, HERK1 and 2, and MIK2 are selected to display their important roles in abiotic stress sensing (Table 2).

FER is the most intensively studied member of the CrRLK1L family. In Arabidopsis, the plasma-membrane-located receptor-like kinase FER, which senses salt-induced cell wall changes, interacts with pectin in the cell wall and elicits salt-induced Ca^2+^ transients to maintain cell wall integrity under salt stress [7]. Loss-of-function *fer* mutants display a significantly lower root growth rate within 24 h treated by salt stress and are unable to fully recover their growth rate after escaping salt stress. Root growth defects in *fer* mutants can only be observed under salt stress but not under hyperosmotic stress, indicating that FER participates in plant responses to sodium ion stress, rather than the associated osmotic stress [7]. Three cell wall leucine-rich repeat extensins, LRX3/4/5, have been found to positively regulate salt tolerance in plants. Their loss-of-function double mutant *lrx34* and triple mutant *lrx345* are hypersensitive to salt stress. Retarded growth is observed in *lrx34* and *lrx345*. These phenotypes are similar with *fer* mutans. Coimmunoprecipitation (Co-IP) assays and an in vitro pull-down assay show that two secretory peptides RALF22/23 interact with LRX3/4/5 and FER, respectively. The overexpression of *RALF22* or *RALF23* leads to retarded growth, increased accumulation of anthocyanin, and hypersensitivity to NaCl in the plant, which is similar to *lrx345* and *fer* mutants. RALF peptides induce the internalization of FER and act as a negative regulator of FER function in salt tolerance [56]. The LRX–RALF–FER module functions in sensing high-salinity-induced cell wall disruptions [7,56].

The cell-wall sensing receptor kinase THE1 plays a vital role in the process of cell elongation and not cell division in the hypocotyl [57]. Mutation of THE1 does not affect seedling growth in the background of wild-type plants but attenuates growth inhibition and ectopic lignification in the background of mutants with the mutation of cellulose synthase CESA6, indicating that THE1 mediates growth inhibition led by defective cellulose synthesis in plants [58]. THE1 is reported as a pH-dependent receptor for RALF34 by micro-scale thermophoresis (MST) assay. The RALF34-THE1 signaling module fine-tunes lateral root initiation in a manner dependent on FER [59].

To investigate whether salt application disturbs other CrRLK1Ls, apart from FER, Gigli-Bisceglia et al. collected six available cell-wall-integrity-sensing-associated mutants from the CrRLK1L protein family, including *the1-1*, *the1-4*, *herk1*, *fer-4*, *the1-1 fer-4,* and *herk1 the1-4*. After 10 days of growth in medium containing 150 mM NaCl, only *the1-4*, *herk1 the1-4*, *fer-4*, and *the1-1 fer-4* displayed significant salt-induced cotyledon bleaching. The single mutant *fer-4* and double mutant *the1-1 fer-4* showed similar rates of cotyledon bleaching. The double mutant *herk1 the1-4* showed enhanced cotyledon bleaching compared with that in the single mutant *the1-4*. These results suggested that FER alone or HERK1/THE1 positively regulates salt tolerance and cell-wall-integrity-dependent salt-sensing mechanisms are complex [60].

HERK1 and 2, THE1, and FER, which act as cell wall integrity sensors, differentially regulate growth adaptation triggered by metal ion stresses [61]. Based on a hypocotyl elongation assay, loss-of-function mutants, *herk1*, *herk2.1*, and *herk2.2* displayed enhanced hypocotyl elongation in response to Cd, Ni, Zn, and Pb. The loss-of-function mutant *the1-6* showed Ni-specific promotion of hypocotyl elongation in the dark. The loss-of-function mutant *fer-4* displayed strong hypocotyl elongation in response to Cd, Cu, Pb, and Zn. Based on a root growth assay, *herk1*, *herk2.1*, and *herk2.2* displayed inhibited root growth in response to Cd, Cu, and Ni. The mutant *the1-6* showed attenuated Cd-specific root growth. The mutant *fer-4* displayed inhibited root growth in response to Ni [61]. These studies indicate the functional diversity of CrRLK1L family proteins in plant response to metal stress.

As a primary component in cell-wall-integrity sensing, MIK2 links cell-wall-integrity sensing to plant development and environmental acclimation. The structure of MIK2 protein contains three domains: an extracellular domain consisting of 24 LRRs, a single-pass transmembrane domain, and an intracellular kinase domain [62]. When grown vertically on MS medium, *mik2-1* and *mik2-2*, two loss-of-function mutants of *MIK2*, showedleft-ward root skewing, while *the1-1* and *the1-4* did not. In the background of the *the1-1* mutant, this effect of *mik2-1* was abolished. In addition, two cellulose biosynthesis inhibitors isoxaben (ISX) and 2,6-di-chlorobenzonitrile (DCB) impaired left-ward root skewing in *mik2-1*. These phenotypes suggest that MIK2 regulates root angle by a THE1- and cellulose synthase-dependent manner. Furthermore, *mik2-1* and *mik2-2* are sensitive to salt stress, while *the1-1*, *the1-4*, and *mik2-1 the1-1* did not, indicating that MIK2 improves salt tolerance in a THE1-dependent manner [62].

#### 2.2.2. HPCA1

As an important second messenger, ROS also plays a key role in abiotic stress sensing. The major forms of ROS include hydrogen peroxide (H_2_O_2_), superoxide (O_2_^−^), singlet oxygen (^1^O_2_), and the hydroxyl radical (HO) based on their properties and chemical reactivity [65]. Recently, considerable attention has been given to H_2_O_2_ because of its prominent role in the regulation of biological activity in cells during the lifecycle of plants [66,67,68]. GUARD CELL HYDROGEN PEROXIDE-RESISTANT1 (GHR1) can monitor H_2_O_2_ signaling and mediate ABA- and H_2_O_2_-regulated stomatal movement in Arabidopsis [69]. However, the mechanism underlying the initial sensing of H_2_O_2_ remains poorly understood. In Arabidopsis, *HPCA1* is a novel extracellular H_2_O_2_ sensor encoding a leucine-rich repeat receptor kinase located in the plasma membrane (Table 2) [10]. The *hpca1* mutant is defective in extracellular H_2_O_2_-induced Ca^2+^ influx and ABA signaling pathways in guard cells, leading to reduced stomatal closure. H_2_O_2_ activates HPCA1 via covalent modification of extracellular cysteine residues in the extracellular domain of HPCA1, leading to autophosphorylation of HPCA1 and subsequent activation of plasma membrane-localized Ca^2+^ channels [10]. However, HPCA1-gated Ca^2+^ channels in plants remain to be identified.

#### 2.2.3. RGFRs and PEPRs

Extracellular pH plays an important role in regulating various biological processes in plants, such as nutrient uptake, cell-to-cell communication, and plant–microbe interactions [70]. The identification of the plant cell surface peptide-receptor complexes, including RGF1-RGFRs and Pep1-PEPRs, represents a significant breakthrough in understanding how plants sense extracellular pH (Table 2) [63]. RGFRs and PEPRs belong to the leucine-rich repeat receptor kinase family. The acidic extracellular pH in the root apical meristem (RAM) region is alkalinized by pattern-triggered immunity (PTI). The interaction between RGF1 and its receptors (RGFRs), which regulate RAM growth, is acid-dependent and inhibited by extracellular alkalinization through the pH sensor sulfotyrosine, while the binding of plant elicitor peptides (Peps) to its receptors (PEPRs) is alkaline-dependent and promoted by extracellular alkalinization through the pH sensor Glu/Asp, which promotes immunity [63]. However, whether plant cell-surface peptide-receptor complexes sense abiotic-stress-triggered extracellular alkalization needs to be elucidated in further studies.

#### 2.2.4. ALR1

Recently, another LRR receptor-like kinase, ALR1, was identified as a plant aluminum (Al) ion sensor (Table 2) [64]. As a highly phytotoxic ion, Al at very low micromolar concentrations can cause cellular damage and inhibit root growth, leading to a severe reduction in crop production [71]. To reduce Al toxicity, ALR1 specifically binds to Al ions through the intracellular cytoplasmic domain, recruits its coreceptor kinase BAK1, and promotes ALR1-dependent phosphorylation of the NADPH oxidase RbohD, thereby increasing ROS generation. In turn, ROS oxidatively modifies the RAE1 F-box protein. Subsequently, the RAE1-dependent proteolysis of STOP1 is inhibited to activate organic acid anion secretion to detoxify Al. A functional analysis of ALR has provided novel insights into ion-sensing mechanisms in living organisms [64].

Moreover, there are several plant RLKs reported to sense temperature changes and function as an indispensable component in response to extreme temperature stress. A novel calcium/calmodulin-regulated receptor-like cytoplasmic kinase CRLK1 and its paralog CRLK2 positively regulate chilling and freezing tolerance [72,73]. The plasma-membrane-localized RLCK, cold-responsive protein kinase 1 (CRPK1), interacts with and phosphorylates 14-3-3 proteins to reduce freezing tolerance in plants [74]. Shen et al. found that the receptor-like kinase ERECTA (ER) performs a vital role in conferring thermotolerance in *Arabidopsis thaliana*, rice, and tomato [75]. Thermo-Sensitive Genic Male Sterile 10 (TMS10) and its close homolog TMS10-Like (TMS10L), which encode two rice leucine-rich repeat–receptor-like kinases, mediate tapetal degeneration and male fertility by buffering environmental temperature changes [76].

### 2.3. Sphingolipids

As one of three main classes of lipids in eukaryotic plasma membranes, sphingolipids are required for preserving normal cellular functions [77]. In plants, sphingolipids are divided into four classes: free long-chain bases (LCBs), ceramides, glycosylceramides, and glycosylinositol phosphoceramides (GIPCs). GIPCs are the major class of plant sphingolipids (64% of total sphingolipids) and represent ~25% of the plasma membrane lipids in Arabidopsis leaves and ~40% in tobacco leaves [78,79]. GIPC has been proposed to be a bioactive molecule involved in cell wall anchoring, cell surface recognition, and lipid-mediated protein anchoring [77,80,81]. To date, there is little information about the precise roles of GIPCs in plants.

A recent study confirmed the possibility of sphingolipid function in abiotic stress sensing in plants. In Arabidopsis, Jiang and colleagues reported that plant cell-surface GIPCs function as a salt stress sensor (Table 3) [8]. Based on screening for salt-stress-induced Ca^2+^ transients, *monocation-induced [Ca^2+^]_i_ increase 1* (*MOCA1*), which encodes an inositol phosphorylceramide glucuronosyltransferase (IPUT1), was identified to be required for [Ca^2+^]_i_ increase. IPUT1 resides on plasma membranes and ER membranes and catalyzes the biosynthesis of the sphingolipid glycosyl inositol phosphorylceramide (GIPC). The *moca1* mutant plant exhibits reduced Ca^2+^ spikes initiated by monovalent cations (Na^+^ as well as K^+^ and Li^+^) but exhibits no change in the Ca^2+^ spikes initiated by oxidative stress (caused by high concentrations of H_2_O_2_), cold stress, osmotic stress (caused by high concentrations of sorbitol), or multivalent cations. Interestingly, the growth of *moca1* seedlings is inhibited by Na^+^ but not by K^+^ or Li^+^. Isothermal titration calorimetry (ITC) analyses revealed that Na^+^ ions bind to GIPCs, which induces depolarization of the cell membrane. MOCA1-dependent GIPC senses changes in extracellular Na^+^ concentrations and leads to salt-dependent intracellular Ca^2+^ spikes via unknown Ca^2+^ transporter(s) [8]. The Ca^2+^-permeable transporters AtANN1 and AtANN4 may be candidate GIPC-gated Ca^2+^ transporters whose mutation leads to a disrupted salt-stress-induced Ca^2+^ signature [41,42].

### 2.4. Other Proteins

In addition to Ca^2+^-permeable channels, RLKs, and sphingolipids, other proteins are involved in abiotic stress sensing. Because of their diverse protein structures, they are assigned to a fourth group, named “Other proteins”, which are involved in sensing extreme temperature stress, mechanical stress, and hypoxia stress.

#### 2.4.1. Extreme Temperature Stress Sensors

With respect to extreme temperature stress, some proteins sense only cold stress or heat stress, such as chilling tolerance divergence 1 (COLD1), ELF3, THERMO-WITH ABA-RESPONSE 1 (TWA1), and heat shock proteins (HSPs), while some proteins can sense cold stress and heat stress simultaneously, such as phyB [3,21,22,23,82,83,84,85] (Table 3). In rice, the transmembrane protein COLD1 has been identified as a potential cold sensor that interacts with G-protein α subunit 1 (RGA1) to activate Ca^2+^ channels and enhance rice cold tolerance [3]. As a thermosensor in Arabidopsis, the nuclear protein ELF3 negatively regulates elevated temperature tolerance. ELF3 contains a polyglutamine (polyQ) repeat embedded within a predicted prion domain (PrD). ELF3-GFP forms speckles within minutes in response to higher temperatures in a PrD-dependent manner. In vitro, the ELF3 PrD reversibly formed liquid droplets in response to temperature, reflecting a direct biophysical response conferred by the PrD. The ability of temperature to rapidly shift ELF3 between active and inactive states occurs via phase transition [21]. Recently, TWA1 has been found to function as a temperature sensor that is required for basal and acquired thermotolerance in Arabidopsis [82]. TWA1 is a transcriptional co-regulator. At elevated temperatures, TWA1 accumulates in nuclear subdomains, changes its conformation, and physically interacts with JASMONATE-ASSOCIATED MYC-LIKE (JAM) transcription factors and TOPLESS (TPL) and TOPLESS-RELATED (TPR) proteins, triggering transcriptional upregulation of the heat shock transcription factor A2 (HSFA2) and HSPs [82]. HSPs have been reported to participate in high-temperature perception. Heat stress leads to protein denaturation and misfolded proteins, causing protein aggregation, which is sensed by HSPs. When HSPs bind to aggregated proteins, heat shock factor (HSF) transcription factors are released to activate heat stress responses [83,84]. In addition, the canonical photoreceptor phyB, which is also implicated as a temperature sensor, plays an important role in plant responses to both warm and cold temperatures through its temperature-dependent reversion from the active Pfr state (a far-red-light-absorbing form) to the inactive Pr state (a red-light-absorbing form) [85]. Under cold stress, the stabilization of phyB is induced via the accumulation of the key transcription factor C-REPEAT BINDING FACTOR (CBF), which interacts with PHYTOCHROME-INTERACTING FACTOR 3 (PIF3) to attenuate the mutually assured destruction of PIF3–phyB. Cold-stabilized phyB enhances freezing tolerance in Arabidopsis [22]. Under elevated temperatures, the phyB Pfr-to-Pr reversion is facilitated to release the inhibition of the Pfr dimer on PIF4 and PIF7 and subsequently activate downstream responses [23].

#### 2.4.2. Mechanosensitive Sensors

Mechanosensitive ion channels of small conductance (MscS)-like proteins (MSLs) and two-pore potassium (TPK) family proteins have been identified as mechanosensitive sensors [86,87] (Table 3). In Arabidopsis, MscS-like 8 (AtMSL8) has been identified as a sensor of hypo-osmotic-stress-induced membrane tension in pollen [86]. AtMSL8 is a pollen-specific, membrane-tension-gated ion channel and decreases the survival rates of pollen grains exposed to the hypo-osmotic shock of rehydration. The hypo-osmolarity induced increases membrane tension and leads to the opening of MSL8, allowing ion efflux, which protects the cells from internal osmotic pressure [86]. Plant TPKs play an important role in vacuolar K^+^ homeostasis and are regulated by Ca^2+^ and 14-3-3 proteins. In Arabidopsis, rice, and barley, vacuolar TPKs can act as intracellular osmosensors via the detection of small perturbations in membrane tension and rapidly increase channel activity during hypo-osmotic shock to release vacuolar K^+^ [87].

#### 2.4.3. Hypoxia Stress Sensors

O_2_ sensors play an important role in plant perception to hypoxia stress (oxygen depletion) induced by flooding. O_2_ sensing in plants is mediated by an N-end rule pathway for protein destabilization. In Arabidopsis, the N-end rule pathway of targeted proteolysis acts as severe low oxygen sensor, such as the hypoxia-associated Ethylene Response Factor (ERF) Group VII transcription factors (ERFVIIs) destabilization (Table 3) [88,89]. All ERFVIIs in Arabidopsis contain a conserved amino-terminal amino acid sequence MCGGAIIL to be dedicated to an oxygen-dependent sequence of post-translational modifications. The Met in this conserved sequence is removed by MET AMINO-PEPTIDASE (MetAP), exposing the destabilizing Cys at the N terminus and leading to the initiation of degradation of ERFVIIs under aerobic conditions. During hypoxia, the loss of oxidation of the N terminus of ERFVIIs maintains ERFVIIs stabilization, and then, ERFVIIs migrate to the nucleus and regulate hypoxia-responsive gene expression [88,89,90]. In addition, the oxidases PLANT CYS OXIDASE 1 (PCO1) and PCO2 are also considered as O_2_ sensors because O_2_ is the direct ligand of PCO1/2 (Table 3) [91].

## 3. Conclusions

**Figure 1:**
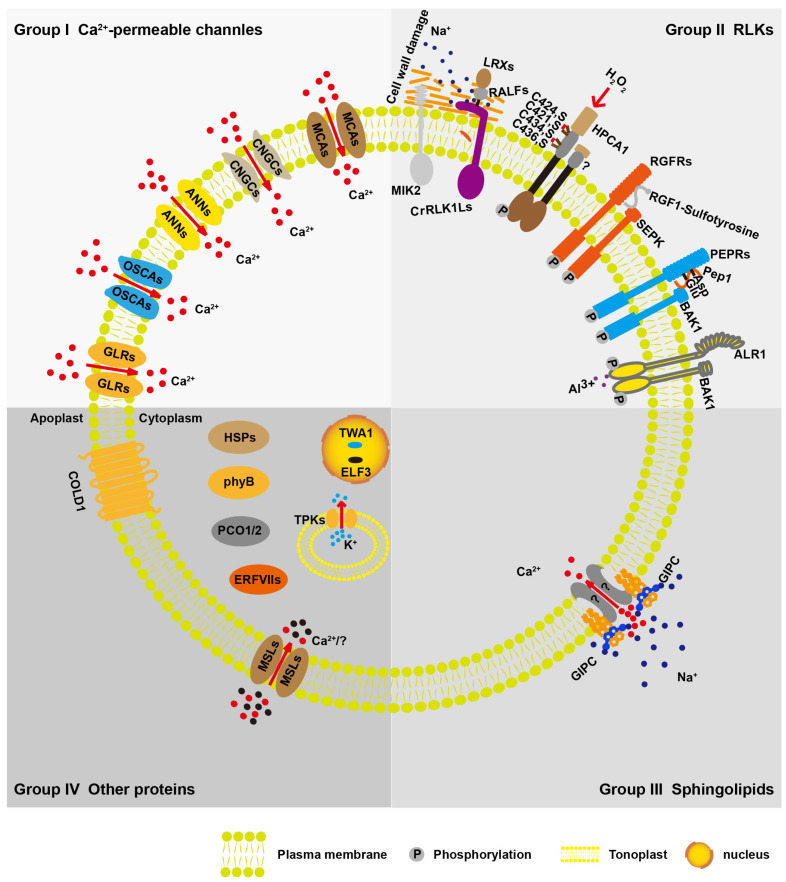
Summary of discussed biomolecule function in abiotic stress sensing. Based on molecular structure, the biomolecule function in abiotic stress sensing can be divided into four groups. Group I: Ca^2+^-permeable channels, including hyperosmolarity-gated calcium-permeable channel family of proteins (OSCAs), cyclic-nucleotide-gated calcium channels (CNGCs), ANNEXIN proteins (ANNs), glutamate receptor-like proteins (GLRs), and MID1-COMPLEMENTING ACTIVITY proteins (MCAs); Group II: receptor-like kinases (RLKs), including *Catharanthus roseus* receptor-like kinase 1-like family proteins (CrRLK1Ls), such as FERONIA (FER), THESEUS1 (THE1), HERCULES1 and 2 (HERK1 and 2), MALE DISCOVERER1-INTERACTING RECEPTOR LIKE KINASE 2/LEUCINE-RICH REPEAT KINASE FAMILY PROTEIN INDUCED BY SALT STRESS (MIK2/LRR-KISS), hydrogen-peroxide-induced Ca^2+^ increases 1 (HPCA1), root meristem growth factor receptors and plant elicitor peptide receptors (RGFRs and PEPRs), and the aluminum ion sensor Al Resistance1 (ALR1); Group III: sphingolipids, including glycosylinositol phosphoceramides (GIPCs); Group IV: other proteins, including chilling tolerance divergence 1 (COLD1), EARLY FLOWERING 3 (ELF3), THERMO-WITH ABA-RESPONSE 1 (TWA1), heat shock proteins (HSPs), phytochrome B (phyB), mechanosensitive channel of small conductance (MscS)-like proteins (MSLs), and two-pore potassium family proteins (TPKs), ethylene response factor (ERF) group VII transcription factors (ERFVIIs), and PLANT CYS OXIDASE 1/2 (PCO1/2). Na^+^: dark blue dot; K^+^: light blue dot; Ca^2+^: red dot; “?”: unknown ions, black dot.

The mechanisms underlying plant sensors and the sensory systems involved in the detection of environmental abiotic-stress-related stimuli have been explored. In the last decade, major advances have been made in the discovery of abiotic sensors, such as the osmotic stress sensor OSCA1, the temperature stress sensor phyB, the salt stress sensor GIPC, and the oxidation stress sensor HPCA1 (Figure 1). In this review, based on molecular structure, we divide biomolecules participating in abiotic stress sensing into four groups: Ca^2+^-permeable channels, receptor-like kinases (RLKs), sphingolipids, and other proteins (Figure 1). Our classification analysis is helpful for revealing many unknown stress sensors. For example, most reported abiotic stress sensors are calcium (Ca^2+^) channels or regulators of Ca^2+^ influx, suggesting the indispensable role of Ca^2+^ in response to environmental stress. Therefore, further experimental investigations of stress sensors could focus on clarifying the functional relevance of the reciprocal bilateral regulation of stress perception by Ca^2+^.

## 4. Future Perspectives

Many gaps remain in our understanding of plant sensory mechanisms. There are many stress sensors to be identified. Sensing mechanisms of most of the reported stress sensors remain unclear. Crosstalk among these sensors is poorly understood. Future research on abiotic stress sensors must involve two goals. First, we must elucidate the means by which abiotic stress sensors perceive stress. Most reported sensors are also considered putative sensors because of their unclear physiological functions and biochemical sensing mechanisms. Studies to unravel the mode of action of these perception mechanisms are complex, as different sensors in different plant tissues share the same downstream signaling pathway in response to different abiotic stresses and combinations of stresses [7,8,9,10,32]. Second, more new abiotic stress sensors need to be identified. It is believed that more sensing mechanisms exist under environmental stress because the known modes of action in abiotic stress perception cannot account for all observed physiological responses. Genetic redundancy and lethality makes it difficult to identify new abiotic stress sensors [32,92,93]. Molecular genetic methods and various bioimaging techniques, including Ca^2+^ imaging-based forward genetic screens and fluorescence-based Ca^2+^ indicators help scientists find new sensors.

The cultivation of stress-resilient crops with improved yield stability is the most effective strategy for overcoming multiple and fluctuating environmental cues. Natural genetic variation in crops, genetic engineering, chemical intervention, and microbial stimulation are usually used in this strategy. In recent years, several success stories about improving crop stress tolerance have been reported. For instance, *HKT1* alleles in rice, wheat, and maize have been identified as major quantitative trait loci regulating salt tolerance and have enabled marker-assisted breeding of wheat with increased yield in saline soils [2,4,94]. The overexpression of the stress-inducible transcription factor *OsDREB2A* enhances dehydration and salt-stress tolerance in rice [1,95]. The application of ABA mimics reduces water loss and promotes drought resistance in plants [96,97]. In addition, approaches combining genetic, chemical, and microbial tactics could provide a promising strategy for cultivating crops with both high stress resistance and high productivity [97,98]. Knowledge of plant sensory mechanisms under abiotic stresses will help us to identify more key molecular targets for engineering stress-resilient crops in the field.

## Figures and Tables

**Table 1:** List of discussed Ca^2+^-permeable channels involved in plant sensing of abiotic stresses.

Groups	Type	Species	Biomolecule Names	Functions	References
I: Ca^2+^-permeable channels	Hyperosmolarity-gated calcium-permeable channel family of proteins (OSCAs)	*Arabidopsis thaliana*	AtOSCA1	Hyper-osmosensors; Regulate primary root length, leaf area, and transpiration	[9]
			AtOSCA2.1	Hypo-osmosensors; Regulate pollen germination	[32]
			AtOSCA2.2		
	Cyclic-nucleotide-gated calcium channels (CNGCs)	*Oryza sativa*	OsCNGC9	Tolerance to chilling shock	[15]
			OsCNGC14	Tolerance to extreme temperatures; Regulate H_2_O_2_ accumulation	[17]
			OsCNGC16		
		*Arabidopsis thaliana*	AtCNGC2	Tolerance to extreme temperatures	[17,18]
			AtCNGC4		
			AtCNGC6	Tolerance to heat stress	[19]
	ANNEXIN proteins (ANNs)	*Arabidopsis thaliana*	AtANN1	Tolerance to extreme temperatures and salt stress	[39,40,41]
			AtANN4		[39,40,42]
	Glutamate receptor-like proteins (GLRs)	*Arabidopsis thaliana*	AtGLR3.3	Regulate membrane depolarization	[43]
			AtGLR3.4	Tolerance to touch and cold stress	[44]
	Mid1-complementing activity proteins (MCAs)	*Arabidopsis thaliana*	AtMCA1	Tolerance to mechanical stress	[13,45,46,47]
			AtMCA2		[45,46]

**Table 2:** List of discussed RLKs involved in plant sensing of abiotic stresses.

Groups	Type	Species	Biomolecule Names	Functions	References
II: RLKs	Cell wall integrity sensors	*Arabidopsis thaliana*	FERONIA (FER)	Tolerance to salt stress and metal ion stresses; Maintain cell wall integrity	[7,56,60,61]
			THESEUS1 (THE1)		[57,58,59,60,61]
			HERCULES1 (HERK1)		[60,61]
			HERCULES2 (HERK2)		
			MIK2/LRR-KISS	Tolerance to salt stress; Maintain cell wall integrity	[62]
	Hydrogen-peroxide-induced Ca^2+^ increases 1 (HPCA1)	*Arabidopsis thaliana*	HPCA1	H_2_O_2_ sensor; Tolerance to oxidative stress; Regulates stomatal movement	[10]
	Root meristem growth factor receptors and plant elicitor peptide receptors (RGFRs and PEPRs)	*Arabidopsis thaliana*	RGFR1	Sense extracellular pH in plants; Promote plant immunity	[63]
			RGFR4		
			PEPR1		
			PEPR2		
	Aluminum ion sensor Al Resistance1 (ALR1)	*Arabidopsis thaliana*	ALR1	Reduces Al toxicity; Regulates ROS generation	[64]

MIK2/LRR-KISS: male discoverer1-interacting receptor like kinase 2/leucine-rich repeat kinase family protein induced by salt stress.

**Table 3:** List of discussed sphingolipids and “other proteins” involved in plant sensing of abiotic stresses.

Groups	Type	Species	Biomolecule Names	Functions	References
III: Sphingolipids	Glycosylinositol phosphoceramides (GIPCs)	*Arabidopsis thaliana*	GIPCs	Salt stress sensor	[8]
IV: Other proteins	Extreme temperature stress sensors *Arabidopsis thaliana*		Chilling tolerance divergence 1 (COLD1)	Tolerance to chilling shock	[3]
			EARLY FLOWERING 3 (ELF3)	Tolerance to heat stress	[21]
			THERMO-WITH ABA-RESPONSE 1 (TWA1)	Tolerance to heat stress	[82]
			Heat shock proteins (HSPs)	Tolerance to heat stress	[83,84]
			Phytochrome B (phyB)	Tolerance to extreme temperatures	[22,23,85]
	Mechanosensitive sensors	*Arabidopsis thaliana*	AtMSL8	Tolerance to mechanical stress	[86]
			AtTPK1		[87]
		*Hordeum vulgare*	HvTPK1		
		*Oryza sativa*	OsTPKa		
	Hypoxia stress sensors	*Arabidopsis thaliana*	Ethylene Response Factor (ERF) Group VII transcription factors (ERFVIIs)	Tolerance to hypoxia stress	[88,89,90]
			PLANT CYS OXIDASE 1 (PCO1)		[91]
			PCO2		
